# Barriers to and Facilitators of the Prescription of mHealth Apps in Australian General Practice: Qualitative Study

**DOI:** 10.2196/17447

**Published:** 2020-07-30

**Authors:** Oyungerel Byambasuren, Elaine Beller, Tammy Hoffmann, Paul Glasziou

**Affiliations:** 1 Institute for Evidence-Based Healthcare Bond University Robina Australia

**Keywords:** mobile apps, mHealth, apps, app prescription, general practice

## Abstract

**Background:**

The ubiquity of smartphones and health apps make them a potential self-management tool for patients that could be prescribed by medical professionals. However, little is known about how Australian general practitioners and their patients view the possibility of prescribing mobile health (mHealth) apps as a nondrug intervention.

**Objective:**

This study aimed to determine barriers and facilitators to prescribing mHealth apps in Australian general practice from the perspective of general practitioners and their patients.

**Methods:**

We conducted semistructured interviews in Australian general practice settings with purposively sampled general practitioners and patients. The audio-recorded interviews were transcribed, coded, and thematically analyzed by two researchers.

**Results:**

Interview participants included 20 general practitioners and 15 adult patients. General practitioners’ perceived barriers to prescribing apps included a generational difference in the digital propensity for providers and patients; lack of knowledge of prescribable apps and trustworthy sources to access them; the time commitment required of providers and patients to learn and use the apps; and concerns about privacy, safety, and trustworthiness of health apps. General practitioners perceived facilitators as trustworthy sources to access prescribable apps and information, and younger generation and widespread smartphone ownership. For patients, the main barriers were older age and usability of mHealth apps. Patients were not concerned about privacy and data safety issues regarding health app use. Facilitators for patients included the ubiquity of smartphones and apps, especially for the younger generation and recommendation of apps by doctors. We identified evidence of effectiveness as an independent theme from both the provider and patient perspectives.

**Conclusions:**

mHealth app prescription appears to be feasible in general practice. The barriers and facilitators identified by the providers and patients overlapped, though privacy was of less concern to patients. The involvement of health professionals and patients is vital for the successful integration of effective, evidence-based mHealth apps with clinical practice.

## Introduction

The number of smartphones and mobile health (mHealth) apps has risen globally, with Australians at the forefront with smartphone ownership near 90% of the population [1]. In addition to fitness and wellness, mHealth apps are primarily created for and can benefit patients in managing chronic diseases [2]. More than half of US consumers have downloaded at least one mHealth app [3]. Despite the high initial uptake of apps, user retention rates can be low, and the duration of app usage can be short [4,5]. However, according to AppScript, an app prescription platform, prescribed mHealth apps have a higher retention rate than nonprescribed apps [2].

Health professionals prescribe mHealth apps in their practice in varying degrees [6-9]. Although relevant professional organizations provide some guidance on how to prescribe mHealth technology in clinical practice, health professionals are often left to navigate this new area on their own [10-12]. A systematic review by Gagnon et al [13] identified about 180 individual barriers and facilitators to adoption of mHealth by health professionals, about third of which reflect factors directly relevant to their knowledge, attitude toward, and acceptance of mHealth. However, these findings were not specific to general practice.

In Australia, the Royal Australian College of General Practitioners (RACGP) offers limited guidance on mHealth apps. The college also has been collecting basic data on providers’ app usage as part of their Annual Technology survey [14]. However, the survey has not explored potential barriers to mHealth app adoption in-depth. It is essential to explore the issues around app prescription in general practice in order to devise effective interventions to overcome the barriers perceived by the practitioners. Therefore, the objectives of this study were to determine the barriers to and facilitators of the prescription of mHealth apps in Australian general practice from the perspectives of general practitioners and their patients.

## Methods

### Participants

We conducted one-to-one semistructured interviews with 20 Australian general practitioners (GPs; 8 via telephone and 12 face-to-face) and 15 patients (all face-to-face) in South East Queensland (Australia) general practices between July and December 2017. We recruited the participants using purposive sampling to ensure a diverse range of years of experience and age. Recruitment was done mainly through snowballing via colleagues, organizational contacts, and via initial participants. Participation in the interviews was voluntary and written informed consent was obtained from each participant before the interview. GPs were interviewed in their consultation rooms or over the phone. Patients were interviewed in the waiting area of the clinic if privacy was ensured.

### Procedure

**Figure 1:**
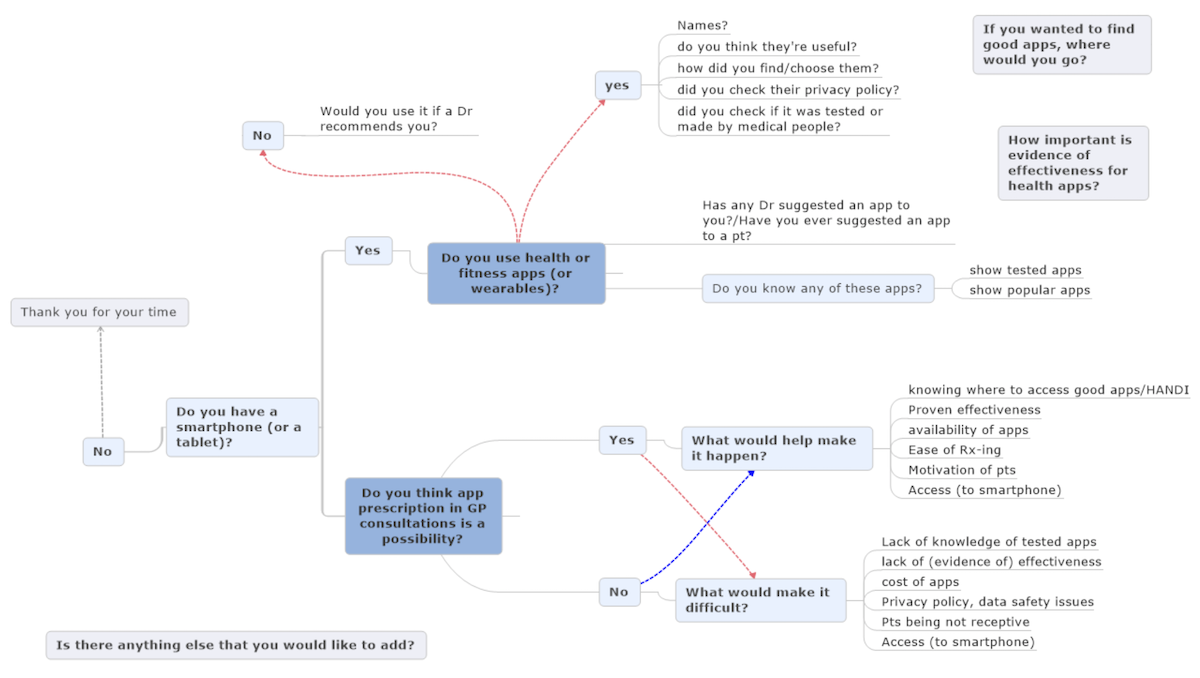
Guide map for the interview.

**Figure 2:**
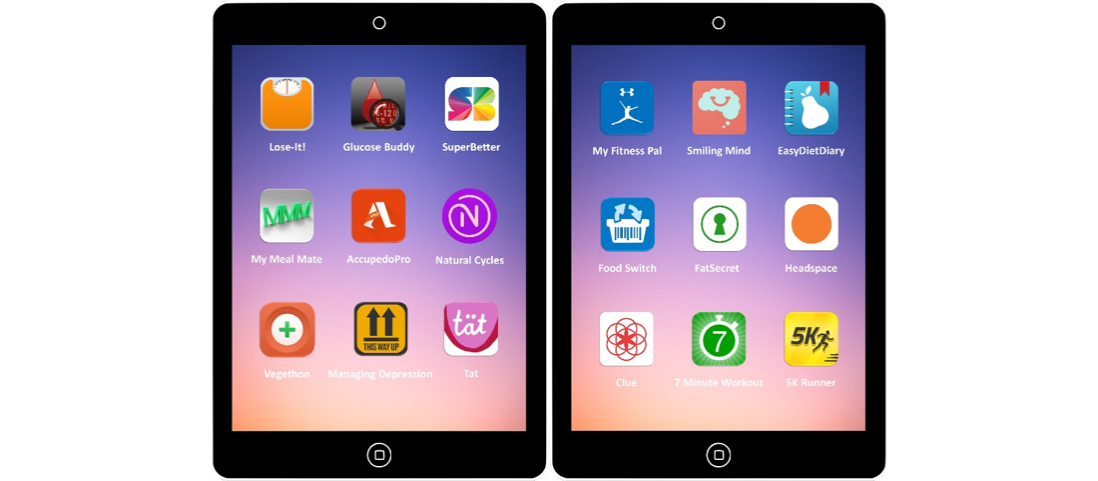
Example apps are shown to interviewees to identify their familiarity with evidence-based vs. popular mHealth apps.

We chose semistructured interviews as they allow for flexibility to explore a new subject yet are structured enough to achieve the study aim. The interview questions were developed by the authors, piloted with three academic GPs, and revised before the study. The questions were designed to explore participants’ attitudes toward smartphone health apps, their thoughts on the possibility of prescribing health apps, and perceived potential barriers and facilitators to prescribing apps in general practice consultations (Figure 1). Toward the end of the interview, participants were shown (or in the case of phone interviews, apps were named) 9 examples of popular Health & Fitness and Medical apps from the major app stores and 9 examples of tested and effected apps identified through our earlier study on the evidence supporting health and medical apps in order to gauge their general familiarity with mHealth apps [15] (Figure 2). No financial or other incentives were offered to participants.

### Data Analysis

We planned to interview 15-20 GPs and a similar number of patients. Data saturation was fully achieved, ie, no new content emerged after 3 consecutive interviews in both cohorts by the time we interviewed 20 GPs and 15 patients, therefore validating that our sample size was sufficient. All interviews were conducted, audio-recorded, and transcribed verbatim by the first author (OB).

We employed a thematic analysis methodology described by Braun and Clarke [16]. The six phases of analysis were familiarization with the data, coding, generating initial themes, reviewing themes, defining and naming themes, and report writing. Two researchers (OB, RS) familiarized themselves with the interview transcripts and independently coded them. Partially inductive thematic analysis was carried out, and generated themes were reviewed in consultation with the third author (TH). The results are written up by the first author (OB).

We used the Consolidated Criteria for Reporting Qualitative Research checklist to report the details of our study [17], which is provided as Multimedia Appendix 1. Ethics approval was obtained from the Bond University Human Research Ethics Committee (16016).

## Results

We interviewed 20 general practitioners and 15 patients, the demographics of whom are shown in Table 1. Ten additional patients declined to be interviewed, citing that they have never used mHealth apps or do not know anything about them. None of the providers we approached declined to be interviewed. Interviews averaged about four minutes for patients and twelve minutes for GPs.

**Table 1:** Participant characteristics.

Group				Total, n (%)
**General practitioners (n=20)**				
	**Years in practice**			
		<10	6 (30)	
		10-20	6 (30)	
		>20	8 (40)	
	Gender (female)		12 (60)	
**Patients (n=15)**				
	**Age (years)**			
		18-35	5 (33.3)	
		36-50	5 (33.3)	
		Over 50	5 (33.3)	
	Gender (female)		10 (66.6)	

### General Practitioners’ Perspectives

The majority of the GPs reported using health apps personally, professionally, and recommending health apps to patients. However, most GPs suggest apps mostly in generic terms in areas such as mindfulness and period-tracking without naming specific apps and let patients make the final choice because they do not know any specific vetted and safe apps.

The GPs were more familiar with popular apps such as MyFitnessPal, Headspace, and Smiling Mind than evidence-based apps. None of the GPs was familiar with five of the evidence-based apps (SuperBetter, MyMealMate, AccupedoPro, Vegethon, Tat). Sources for finding health apps to recommend varied from trusted websites and medical publications to word-of-mouth and app stores.

About half of the participants were unfamiliar with the Handbook of Non-Drug Interventions (HANDI) web resource from the RACGP [18], but upon being introduced to it, they all agreed that HANDI would be a trustworthy resource for evidence-based apps, consistent with those GPs who were already familiar with HANDI.

#### Barriers

We identified four themes around barriers to app prescription in general practice. The most prominent barrier cited was a generational difference in the overall digital propensity for both doctors and their patients.

Most patients who would benefit from them are elderly and they don’t do apps. They don’t do smartphones. That’s the number one barrier. And most of our patients who are over 70 will be in that category. Most I would say.

GP7

Well, it’s probably my age group more than anything. it’s just I’m not as familiar with and as happy around technology as the newer doctors… I do feel it’s important to try to learn it because it’s part of the future… so it’s that technology I think is the main barrier as far as being used to it. I think we need to but it’s just hard to do. Can we get away with it? I guess that’s what’s happening to a lot of older doctors is seeing if we really need to do it or not.

GP5

GPs with more than 20 years of experience appeared more likely to find mHealth apps a “gimmick” and less likely to consider using them in their practice.

I think it would be [possible to prescribe apps]. The trouble is I think they’re a bit of a gimmick. I mean we’ve always had accessories to health. When I was a young doctor people would bring in their calendars which showed when their next periods are. Now they pull out their phones… you find the phone is slower than the calendar because the calendar… is there and you’d be able to see it visually, whereas a phone, they’re pulling it out… trying to find it miss it… then the reception is not good and it takes ages for it to download. So I don’t really find them a step forward. They probably are for the person using them at home. But in the consultation, they’re often not a step forward I find.

GP9

GPs who primarily work with elderly patients would not consider using apps as potential interventions. The “cut-off age” for recommending apps to patients was around 40-60 years.

For somebody like me, there will be obstacles because I don’t really use apps. So, if you’re not comfortable with that sort of things yourself, you have to overcome that to recommend it... Even when I’ve learned about them in an education session, I always forget what they are, because I don’t use them… In fact, the only times I ever recommend apps at all are for young people… they don’t have to be young-young, even in their 30s, a young professional… But I never recommend to people older than that. So, there is obviously a generational issue.

GP3

However, exceptions to the age-based generalization were commonly mentioned, making individual digital propensity a more influential factor than age in deciding to prescribe apps.

I got some elderly patients that don’t use an iPhone too often or an app. But there’s a lot of savvy oldies there too. It probably depends on the patient, their interests.

GP11

GPs also recognize that age is a transient barrier as the younger cohort of patients will age and become patients with chronic diseases.

It is a very good idea and something that can be very useful. But I never know which ones are good to use and which population would be good to use them. I’m sure it will come especially as right now the younger population are the ones who are really into the smartphones and they’ll get older and have chronic medical conditions in the next 20 years I’m sure it will there’s going to be a big space for these apps.

GP16

Another barrier was a lack of knowledge of prescribable apps and a lack of trustworthy sources to access them.

Probably the only other barrier is knowing which apps. And keeping on top of all the apps they become available, how much they cost and all those kinds of things.

GP20

I do. I think it’s something I’m cautious of simply because if it’s not something that I know a lot about then I’m a bit more concerned, you know, I don’t really wanna recommend something I don’t know the full workings of, especially if I’m gonna ask them to buy. There’s so much of me asking them to go on medication until I’m confident that money is money worth spent and the benefit outweighs the cost, then I’m not really willing to do that.

GP2

I think for education it’s really valuable. Ones that I don’t use enough of and they all want more information. Otherwise, they’ll just Google some unreliable search, and so if I’ve got a good place to go to that’s evidence-based, that’s good.

GP18

Another barrier was the time commitment required to learn about apps and integrating them into consultations as well as the time commitment required of the patients to use the recommended app. Before they were willing to expend time to adopt apps into their clinical practice, GPs described needing to be convinced of the benefits of using apps.

And it’s time-consuming to learn about these things. It’s hard enough just keeping up with what medicine is doing without this app and that app etc.

GP1

You’re so busy doing in medicine there’s not a lot of time to go out there and research what might need to be done to create an app or even the ones that are out there. We’re so rushed for time. You’re competing with lots of other demands for your time and energy as well. So that’s a big limiting factor.

GP8

The patients’ motivation would be a big thing. And the time involved in using it would be another big thing for someone who might be busy, for example. I don’t think a lot of people have a lot of time to invest in this type of thing. So, I think your time, availability, and the motivation behind a patient.

GP15

I think for me, it’s just when you are consulting, and you’re busy. To modify the practice of what I’m doing, I have to have a pretty good reason for doing it.

GP6

Another barrier identified was the privacy, safety, and trustworthiness of health apps. The GPs perceived issues around privacy and data safety of health apps as the ultimate responsibility of the patients, since their complete and ongoing safety cannot be guaranteed. Some GPs were aware of how the industry attempts to influence health care.

I personally manage my privacy very, very, very carefully. But I think I leave that to the individual patient... and I think that if they’re going to be using apps, my perception is that they already have made their own personal decisions upon privacy.

GP4

I think that’s every day now. I mean the number of times you get on Google and then they already go okay well you’ve got this many children, and I know that already… that’s the world we live in. I don’t think we’re going to stop that by not using an app when we’re on the internet all the time. So, if it’s demographic information and I think that’s being collected by a lot of people, not just an app, I suppose.

GP20

Also, I guess I’m also very wary of who’s paying for it. So, I guess my general approach to most things is to think if you’re going to pay for it, then hopefully you are bearing the load of what if it’s worth it. If someone else is paying then there’s some hidden agenda there, whether it’s a drug company or someone else who’s gonna benefit of you having their app on your phone and again that comes down to my reticence to recommend something I really don’t know who’s designed it and what’s the purpose of it and who’s benefiting the most.

GP2

#### Facilitators

We identified two main themes around facilitators of mHealth app prescription in general practice. One was a trustworthy source for prescribable apps and information for GPs. The RACGP was the most preferred trustworthy source to vet, endorse, and provide prescribable apps as the majority of the GPs were RACGP members.

Maybe some sort of database of trusted apps that would be recommended for certain conditions or treatment strategies. Having a nice little summary of things that could be used, because of the sheer number of what’s on the market, it’s hard to really make it part of my regular day-to-day routine... Whereas if some organization was to put together a list of you know tried and tested and reliable apps then it would be much easier to say “look, you’re young you’ll you've got the time and the patience for it, let's try an app for this problem, and this is the one we trust.

GP15

I think we probably need to have them reliably approved and researched by our college. I probably wouldn’t be happy to recommend any without the endorsement of the college.

GP11

We need more education on which ones to prescribe and which ones not. We have the NPS which helps us with prescribing medications. So, if there were an organization/body involved with educating GPs on which apps are good and useful and provide the right information and are easy to use, that would be really helpful.

GP16

GPs recognized that near-universal ownership of smartphones, the ubiquity of apps, and younger tech-savvy patients are enablers of app prescription, as this facilitates information accessibility and can sometimes provide alternatives that are more convenient and lower in cost.

I work in an area where there's a lot of young people, and most of these people are … generally pretty switched on. and … I can't imagine a situation where I’m not being able to recommend an app so somebody. This hasn't happened yet.

GP12

Apps are quite a neat way of showing somebody all that information without having a book to give them. It's often a very low-cost solution if your alternatives are costly. And it's very accessible... you don't need to wait for in-hours care, you can be 10 p.m. at night and do some of the work, whether it's treatment or information and knowledge that you're sharing that can all be done at a time that suits a patient. so that's the kind of value of apps I suppose over other resources.

GP20

### Patient Perspective

Two-thirds of the patients have used or use mHealth apps personally. The most commonly used app types were fitness, wellness, and women’s health-related apps (period, ovulation, and pregnancy tracking). Those who did not use apps said the reason was they do not have any need or significant reason to do so. People often chose apps through the recommendations of friends or family, directly from app stores, or through subscribed services such as the local gym. From the list of popular and evidence-based apps, they recognized some of the most popular apps, such as MyFitnessPal and 5K runner, but none of the evidence-based apps.

#### Barriers

From the patients’ perspective, a perceived barrier to using mHealth apps in general practice consultations was older patients.

I don’t think there’d be any problem. But if we’re talking about the elderly, they’re not really very computer-savvy. So, they might find it difficult. I have a number of friends, even older than me; they don’t wanna use smartphones. It’s too much trouble. They just wanna make a phone call and get a text. That’s it.

Pt8

I guess only with the older generation not having smartphones. They would not use apps. So that would really be the only problem. Yeah, I wouldn’t see any other [issue].

P2

Another barrier was poor usability of apps.

I think the ease-of-use has to be paramount. Ease of use is gotta be the big issue. For me, that was the problem. I can’t speak for other people. I mean I’m pretty good with technology, but I just found it very tedious… And I just found it was annoying to put data in. That was the issue with it. So, when it becomes difficult, I didn’t do it.

Pt12

Data safety and privacy issues related to health app use were not a barrier described by the patients interviewed. Patients were more concerned about the loss of financial information than health-related information.

I don’t worry about it too much, privacy stuff, cause I don’t have much to hide. Maybe if someone got on to your phone, they could see your personal information, they usually have like a passcode for private apps anyway.

P13

Me personally, no. data safety… it is what it is. I think there are measures in place to keep it safe. Other people probably don’t have that opinion. I think honestly it is safe enough.

P15

I’m more worried about financial stuff than health that would create a financial loss. if somebody finds out what my blood pressure is, what’s the big deal, right?

Pt8

I don’t worry about it. well, I’m not really putting anything into an app that is that [important]… I mean I don’t know who else in the world cares the day my period came or how big the baby is, I haven’t really put in banking details or anything. I haven’t used any paid apps.

Pt6

#### Facilitators

From the patients’ perspective, the ubiquity of smartphones and apps and young patients were perceived as facilitators of app prescription.

I imagine younger people wouldn’t have any problems.

Pt11

I think it’s quite a common thing now. Everyone has smartphones nowadays. So, it’s an easy way to access it.

Pt1

Most patients expressed that they think mHealth apps can be beneficial, especially when recommended by their doctor and that app prescription by GPs is possible, helpful, and welcome because it would eliminate the challenge of finding health apps for themselves.

Great idea if they’re prescribed from a doctor. I don’t think getting them off the net without advice would be a good thing. I think it needs to be advised or prescribed by someone who knows, and then from there, it can only be a good thing, yeah.

Pt15

Yeah. If my doctor recommended one, I’d probably go with that rather than trying to go from the recommendations on iPhone, you know, the stars, from the app stores. I mean, they are helpful too what other users have found, cause a lot of apps crash and have problems if they’re not maintained and upgraded, but yeah, if a doctor was recommending one, I’d probably use it.

Pt9

If there are multiple people being like, “this is really good. This has helped me with this…” then I’ll actually go have a look. If I like it for more than a week, then I’ll just continue using it. I went through a lot [of apps] before finding the one that wasn’t an effort to use, one that was just easy. I went through probably ten to find one I actually liked, which is a bit annoying. It kind of turns you off that. So, if there was one or two that all doctors recommended, then people would probably more likely to use them.

Pt1

A theme identified from both the GPs and patient perspectives that could not be categorized as a barrier or facilitator was evidence of app effectiveness. GPs expressed they would not want to prescribe apps not supported by evidence, yet they also feel that simple apps do not need high levels of evidence.

…as it applies to anything in medicine, I think it would need a reasonable degree of efficacy to run with it. You know, you can’t just have an app for the sake of having an app.

GP1

… [evidence is] pretty important to officially recommend. Some of it is common sense. Like something to log your blood pressure and mood diary is self-explanatory and makes sense. But for some more complex health apps, you sort of wanna know if there’s good evidence so that it’s reasonably well made. Especially if you’re going to spend money on it.

GP14

Most patients viewed the evidence of app effectiveness to be important in the same way as the standards required of pharmaceutical interventions. Some were not concerned about the effectiveness and preferred personal evaluations and the freedom to make the ultimate decision.

Very important. It’s like anything in health; it’s like medication, if a doctor is gonna recommend something, they have to know it works. Cause it might not be suitable for somebody, and if that person is to use something that’s not suitable, then that could have a bad effect instead of a good one.

Pt13

Definitely, yea. I would want one that has references, the app that I use have medical references they’ve sourced the information. Otherwise, anyone could be sitting home and writing an app.

Pt6

I’m not too worried about that. I just get on and try it, if it works for me then that’s great. If it doesn’t, then I’ll just delete it if it’s not gonna do what I needed it to do for me… I mean, as long as it’s our choice to use them or not. I mean up to us, they can make a recommendation, but if we find it’s not suitable or it doesn’t work, and if it does then great if it makes our life easier, it’s a fast and busy world, so if they think it can help, it’s great.

Pt10

## Discussion

In this study of the barriers to and facilitators of mHealth app prescription in Australian general practice, all patients and GPs recognized that mHealth apps could be beneficial, and app prescription is achievable. From the GPs’ perspective, uptake is hindered by barriers around a generational difference in the digital propensity for both GPs and patients, lack of knowledge of prescribable apps and lack of trustworthy source to access them, time commitments required of the GPs and patients, and privacy, safety, and trustworthiness of health apps.

Both patients and GPs cited the old age of patients as a barrier to app prescription, although also offered examples of exceptions to this age-related division of digital propensity. Annual mobile consumer surveys showing that older age groups have seen the highest increase in smartphone ownership in Australia [19]. Doctors and patients also believed that the ubiquity of smartphones and apps, and young patients are facilitating factors as Australians approach “peak smartphone”—the peak level of usage before the vast majority of consumers to begin actively limiting their phone use [20]. Almost all the interviewed GPs reported using apps personally and professionally; however, they do not recommend specific apps to patients. Instead, they remind patients of the availability of mHealth apps in the general area of focus (eg, mindfulness) should they wish to use them.

The evidence base (weak or strong) for mHealth apps emerged as an important theme as a barrier or facilitator. Patients viewed doctors as a trustworthy source of health apps, and the GPs acknowledged that they needed a trustworthy source for prescribable apps as they have neither the knowledge nor time to find such apps themselves. A national-level professional organization such as the RACGP is well-placed to address this barrier. One of the many resources they provide for GPs is HANDI—a database of effective nondrug interventions, which includes several mHealth apps [18]. However, half of the GPs interviewed in this study were not familiar with or commonly used HANDI, but all agreed that RACGP is the most trusted source for them to access professional and practice-related information and guidance. The majority of participants recognized a few of the most popular apps from app stores and fewer apps from the evidence-based apps list, emphasizing the need for information dissemination about evidence-based apps.

Several comparable studies have explored barriers and facilitators to novel technology adoption in medical practice. Many studies report a lack of education and training as one of the most common barriers that face health care providers in adopting new technology in health care [13,21,22], and our study echoes this. Furthermore, the potential to increase doctors’ time strain and workload are universally common factors of poor uptake of new health technologies [23-25]. Brandt et al found that the experiences of GPs with eHealth support for lifestyle changes were an essential factor in recommending it for their patients [26]. Our findings also emphasize the digital propensity of the health care providers and patients would make a big difference in uptake of mHealth apps. Building on this finding and educating and supporting GPs so they understand the value of new technology such as the potential to save consultation time and keep patients connected and motivated between consultations can help mitigate against these barriers and help them recommend apps to their patients with confidence.

Recent research suggests that individuals with poor self-reported health and low rates of physical activity were the least likely to report downloading and using these health tools [27]; however, patients adhere better to prescribed apps [2] beyond the typical one week of usage [4,5]. Thus, the official prescription of apps by trusted medical practitioners might help increase the uptake of effective health apps among such patients who would benefit the most. Future studies should measure the real-world adherence of the patients following app prescription by health professionals.

The present qualitative study appears to be the first of its kind to explore the perspectives of GPs and patients regarding mHealth app prescription in Australian general practice. The barriers identified in this study were added to the mHealth section of the RACGP Annual Technology survey in 2017 to explore further how they would rank among a national sample of GPs [28]. The question about barriers to app prescription gathered over six hundred responses and the top barriers reflected the central theme identified in this study, which was lack of knowledge of effective apps and lack of trustworthy source to access them, further validating the findings of this study.

Limitations of this study include a small sample size that skewed towards relatively healthy patients from high socioeconomic areas and GPs from the metropolitan area. Although we attempted to mitigate this limitation by purposively sampling participants from a variety of age and work experience, future studies should target patients with long-term medical conditions, those from rural and remote areas, and low socioeconomic areas. Other limitations include lack of triangulation of the results, member checking, a reflection of possible interviewer bias about the potential of apps, and not piloting the interview questions with patients.

### Conclusions

mHealth app prescription appears to be feasible in general practice. The barriers and facilitators we identified from both GPs and patients widely overlapped. The involvement of all stakeholders of consumer mobile technology, medical professionals, and patients is vital in the successful integration of mHealth apps with clinical practice. The findings of this study will inform the development of effective interventions to overcome the identified barriers and help the adoption of health apps to general practice to patients’ benefit.

## Appendix

Multimedia Appendix 1 COREQ checklist.

## Abbreviations

GP: general practitioner
HANDI: Handbook of Non-Drug Interventions
mHealth: mobile health
RACGP: Royal Australian College of General Practitioners
