# Supplementary material for: Barriers to and Facilitators of the Prescription of mHealth Apps in Australian General Practice: Qualitative Study
Source: JMIR Mhealth Uhealth. 2020 Jul 30;8(7):e17447. doi: 10.2196/17447 (PMC7426799; doi:10.2196/17447)
Supplement: Multimedia Appendix 1 [file mhealth_v8i7e17447_app1.pdf]

Supplementary table 1
Consolidated criteria for reporting qualitative studies (COREQ): 32-item checklist

No	Item	Guide questions/description	
**Domain 1: Research team and reflexivity**			
Personal Characteristics			
1.	Interviewer/facilitator	Which author/s conducted the interview or focus group?	First author Oyungerel Byambasuren
2.	Credentials	What were the researcher's credentials? *E.g. PhD, MD*	MD/GP, MMedRes
3.	Occupation	What was their occupation at the time of the study?	PhD candidate
4.	Gender	Was the researcher male or female?	Female
5.	Experience and training	What experience or training did the researcher have?	Master of Medical Research
Relationship with participants			
6.	Relationship established	Was a relationship established prior to study commencement?	No
7.	Participant knowledge of the interviewer	What did the participants know about the researcher? e*.g. personal goals, reasons for doing the research*	Participants were informed about my overall PhD objectives and this interview study aims
8.	Interviewer characteristics	What characteristics were reported about the interviewer/facilitator? e.g. *Bias, assumptions, reasons and interests in the research topic*	In the Discussion, we report that the interviewer was not "pro-apps". She went into this research on health apps as an average smartphone owner of 21st century but not a pro-tech digital enthusiast per se.
**Domain 2: study design**			
Theoretical framework			
9.	Methodological orientation and Theory	What methodological orientation was stated to underpin the study? *e.g. grounded theory, discourse analysis, ethnography, phenomenology, content analysis*	Grounded theory
Participant selection			

10.	Sampling	How were participants selected? *e.g. purposive, convenience, consecutive, snowball*	Purposive and snowball
11.	Method of approach	How were participants approached? e*.g. face-to-face, telephone, mail, email*	Face-to-face and email
12.	Sample size	How many participants were in the study?	20 GPs and 15 patients
13.	Non-participation	How many people refused to participate or dropped out? Reasons?	None of the GPs I approached refused to participate. Ten patients refused due to having no experience and opinion about health apps.
Setting			
14.	Setting of data collection	Where was the data collected? e*.g. home, clinic, workplace*	F2F interviews were done in a GP clinic. Telephone interviews mixed
15.	Presence of non-participants	Was anyone else present besides the participants and researchers?	No
16.	Description of sample	What are the important characteristics of the sample? *e.g. demographic data, date*	We purposively sampled participants to include all ages and work experience levels. Demographic data is reported in table 1 in Results section.
Data collection			
17.	Interview guide	Were questions, prompts, guides provided by the authors? Was it pilot tested?	Yes and yes. Reported in Methods section
18.	Repeat interviews	Were repeat interviews carried out? If yes, how many?	No
19.	Audio/visual recording	Did the research use audio or visual recording to collect the data?	Audio recorded
20.	Field notes	Were field notes made during and/or after the interview or focus group?	Yes
21.	Duration	What was the duration of the interviews or focus group?	GP interviews averaged 12 minutes and patient interviews 4 minutes.

22.	Data saturation	Was data saturation discussed?	Yes. Data was considered saturated when there was no new information emerged in 3 consecutive interviews.
23.	Transcripts returned	Were transcripts returned to participants for comment and/or correction?	No
**Domain 3: analysis and findings**			
Data analysis			
24.	Number of data coders	How many data coders coded the data?	Two
25.	Description of the coding tree	Did authors provide a description of the coding tree?	No
26.	Derivation of themes	Were themes identified in advance or derived from the data?	Derived from the data inductively
27.	Software	What software, if applicable, was used to manage the data?	Microsoft Excel
28.	Participant checking	Did participants provide feedback on the findings?	No
Reporting			
29.	Quotations presented	Were participant quotations presented to illustrate the themes / findings? Was each quotation identified? e.*g. participant number*	Yes, as GPs and Pt (patients)
30.	Data and findings consistent	Was there consistency between the data presented and the findings?	Yes
31.	Clarity of major themes	Were major themes clearly presented in the findings?	Yes
32.	Clarity of minor themes	Is there a description of diverse cases or discussion of minor themes?	Yes
